# Refractory renovascular hypertension secondary to Takayasu arteritis treated with aorto-mesenteric bypass

**DOI:** 10.1093/rap/rkac005

**Published:** 2022-02-07

**Authors:** Daniel Jimenez, Tina Tian, Isaac Gendelman, Payam Salehi

**Affiliations:** 1 Tufts University School of Medicine; 2 Department of Surgery, Tufts Medical Center; 3 Division of Vascular Surgery, Cardiovascular Center, Tufts Medical Center, Boston, MA, USA

Dear Editor, Takayasu arteritis is traditionally seen in young Asian females but is also seen in a worldwide distribution. A recent study found a female-to-male ratio of 12:1, with peak age of onset between 20 and 30 years of age [1]. Here, we present a case of Takayasu arteritis in an unusual patient population with atypical vessel distribution [2].

A 52-year-old Caucasian non-smoker male with a history of renovascular hypertension (RVH), atrophic right kidney and hypopituitarism presented with chest pain radiating to his back. Laboratory work revealed a normal troponin level, elevated D-dimer (0.52 µg FEU/ml), elevated ESR (99 mm/h) and elevated CRP (5.1 mg/dl, normal < 0.8 mg/dl). A CT angiography of the chest/abdomen demonstrated thickening and inflammation of his descending thoracic aorta, abdominal aorta, mesenteric vessels, bilateral iliac and bilateral renal arteries. Further history revealed that he was diagnosed with RVH 10 years before, secondary to right renal artery stenosis. Based on the current CT angiography findings, he was diagnosed with possible Takayasu arteritis.

After rheumatological evaluation, our patient was started on high-dose prednisone and AZA, with improvement in inflammatory markers (CRP 1.2 mg/dl; ESR 19 mm/h). His AZA was then discontinued owing to transaminitis. Unfortunately, he was then lost to follow-up.

The patient re-presented 1 year later with another acute exacerbation (CRP 14.8 mg/dl; ESR 31 mm/h), whereupon he was treated again with high-dose prednisone. Tocilizumab was added, with symptom improvement and inflammatory marker normalization (CRP 0.1 mg/dl; ESR 8 mm/h). However, our patient had challenges with tocilizumab infusions and compliance, resulting in subsequent flare-ups. Follow-up CT angiography obtained while on high-dose prednisone continued to show worsening vasculitis. He underwent magnetic resonance angiography, which confirmed progression of large vessel vasculitis and near-critical stenosis of the coeliac, superior mesenteric, inferior mesenteric and bilateral renal arteries.

Given his young age and continued progression, multidisciplinary discussions determined that surgical bypass would be the next best step. Pre-operatively, he was not in an acute inflammatory state (CRP 0.1 mg/dl; ESR 4 mm/h) and had received tocilizumab 3 weeks before. This bypass was performed with a bifurcated synthetic graft (Fig. 1A). Intra-operatively, he was noted to have significantly friable vessels. The proximal graft was anastomosed to the supracoeliac aorta. One graft limb was anastomosed to the coeliac trunk base (Fig. 1B). The second limb was tunnelled to the superior mesenteric artery (Fig. 1C). The left renal artery was ligated from the aorta and anastomosed to the graft. The final anastomosis was performed between the superior mesenteric artery and the graft (Fig. 1D). The patient tolerated the procedure well. Post-operatively, he continued to improve, with down-trending creatinine levels, and was weaned off his three anti-hypertensive medications.

**Fig. 1:**
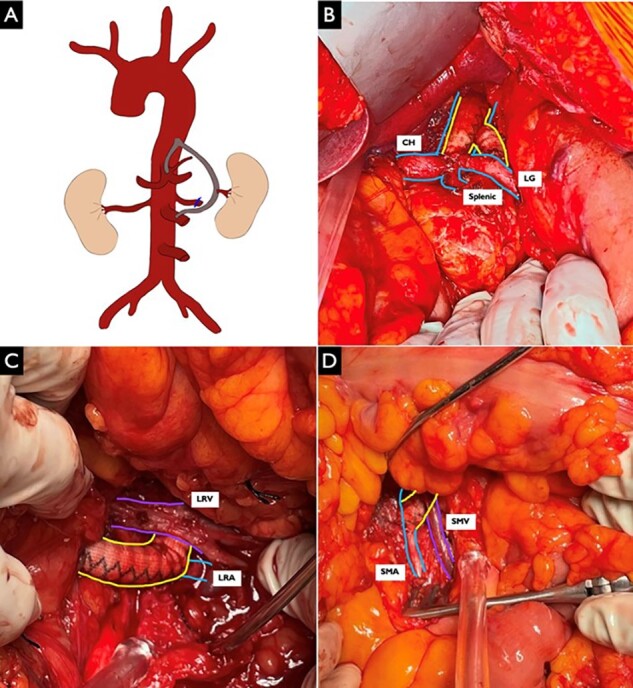
Aorto-coeliac, aorto-superior mesenteric artery and aorto-left renal bypass performed using a 14 mm × 7 mm bifurcated Dacron graft (**A**) Schematic diagram of bypass. (**B**) The proximal portion of the graft (yellow) was anastomosed to the supracoeliac aorta (blue) in an end-to-side fashion. One limb was then anastomosed in an end-to-side fashion to the base of the coeliac trunk. (**C**) The second 7 mm limb was then tunnelled in a retropancreatic fashion, crossing underneath the left renal vein (purple). The left renal artery was anastomosed to the wall of the graft (yellow) in an end-to-side fashion (blue). (**D**) The third and final anastomosis between the superior mesenteric artery (blue) and the graft (yellow) was performed in an end-to-side fashion. CH: common hepatic artery; LG: left gastric artery; LRA: left renal artery; LRV: left renal vein; SMA: superior mesenteric artery; SMV: superior mesenteric vein.

In retrospect, the initial presentation of Takayasu arteritis in this patient was probably the RVH diagnosed 10 years prior. At that time, the patient did not exhibit any additional signs or symptoms of Takayasu arteritis. In Takayasu arteritis patients, it is not uncommon to have renal artery involvement, with upwards of 60% involvement in Asia, which can result in RVH in 33–80% of cases [3]. Often, this RVH becomes refractory to multimodal medical therapy.

Historically, arterial angiography has been the gold standard for diagnosis [4]. However, CT angiography and magnetic resonance angiography have become used increasingly. The main imaging findings are mural thickening and luminal changes. Mural thickening commonly appears as a double ring, with the inner ring representing intimal swelling and the outer ring representing active inflammation in the medial and adventitial layers [5]. Mural thickening is commonly associated with stenosis, seen in 90% of Takayasu arteritis patients. The most commonly stenosed vessels are the thoracic and abdominal aorta, subclavian, common carotid and renal arteries. Additionally, there have been reports of aortic vessel dissections, but not of coeliac or common iliac artery dissection like our patient [6].

Given this patient’s young age and worsening disease on escalating medical therapy, he was evaluated for surgical intervention. Open bypass surgery was chosen over stenting owing to increased risk of restenosis. The higher rate of post-operative restenosis in endovascular approaches is secondary to progression of the native disease process and placement of a foreign body directly at the site of inflammation [7]. In a comparison of open *vs* endovascular interventions for Takayasu arteritis, the 10-year primary patency rate was 48.8% for open surgical and 31.8% for endovascular [7]. Inflammatory markers were normalized for our patient before surgical intervention, because performing reconstructive procedures during active vasculitis has been shown to increase complications such as anastomotic dehiscence, restenosis rates and disease progression in other arterial areas [8].

In conclusion, we present a case of refractory Takayasu arteritis in an atypical patient with unusual vessel involvement, managed with surgical bypass. The progression of this patient’s disease is multifactorial, and the lack of compliance with treatment is likely to have played a substantial role in disease progression. This unique case illustrates the successful use of a triple bypass (aorto-coeliac, aorto-superior mesenteric artery and aorto-renal) for the treatment of refractory RVH secondary to Takayasu arteritis.

*Funding:* No specific funding was received from any bodies in the public, commercial or not-for-profit sectors to carry out the work described in this article.

*Disclosure statement:* None of the authors have any financial conflicts of interest in any of the products, devices or drugs mentioned in this manuscript.

## Data availability statement

Data are available upon reasonable request by any qualified researchers who engage in rigorous, independent scientific research, and will be provided following review and approval of a research proposal and Statistical Analysis Plan (SAP) and execution of a Data Sharing Agreement (DSA). All data relevant to the study are included in the article.
